# Removal ratio of gaseous toluene and xylene transported from air to root zone via the stem by indoor plants

**DOI:** 10.1007/s11356-016-6065-y

**Published:** 2016-01-22

**Authors:** K. J. Kim, H. J. Kim, M. Khalekuzzaman, E. H. Yoo, H. H. Jung, H. S. Jang

**Affiliations:** 1 Urban Agriculture Research Division, National Institute of Horticultural and Herbal Science, Rural Development Administration, Wanju, 560-852 Korea; 2 Department of Genetic Engineering and Biotechnology, University of Rajshahi, Rajshahi, 6205 Bangladesh

**Keywords:** Indoor air, Toluene, Xylene, Plants, Removal, Ratio transportation

## Abstract

This work was designed to investigate the removal efficiency as well as the ratios of toluene and xylene transported from air to root zone via the stem and by direct diffusion from the air into the medium. Indoor plants (*Schefflera actinophylla* and *Ficus benghalensis*) were placed in a sealed test chamber. Shoot or root zone were sealed with a Teflon bag, and gaseous toluene and xylene were exposed. Removal efficiency of toluene and total xylene (*m*, *p*, *o*) was 13.3 and 7.0 μg·m^−3^·m^−2^ leaf area over a 24-h period in *S. actinophylla*, and was 13.0 and 7.3 μg·m^−3^·m^−2^ leaf area in *F. benghalensis*. Gaseous toluene and xylene in a chamber were absorbed through leaf and transported via the stem, and finally reached to root zone, and also transported by direct diffusion from the air into the medium. Toluene and xylene transported via the stem was decreased with time after exposure. Xylene transported via the stem was higher than that by direct diffusion from the air into the medium over a 24-h period. The ratios of toluene transported via the stem versus direct diffusion from the air into the medium were 46.3 and 53.7 % in *S. actinophylla*, and 46.9 and 53.1 % in *F. benghalensis*, for an average of 47 and 53 % for both species. The ratios of *m*,*p*-xylene transported over 3 to 9 h via the stem versus direct diffusion from the air into the medium was 58.5 and 41.5 % in *S. actinophylla*, and 60.7 and 39.3 % in *F. benghalensis*, for an average of 60 and 40 % for both species, whereas the ratios of *o*-xylene transported via the stem versus direct diffusion from the air into the medium were 61 and 39 %. Both *S. actinophylla* and *F. benghalensis* removed toluene and xylene from the air. The ratios of toluene and xylene transported from air to root zone via the stem were 47 and 60 %, respectively. This result suggests that root zone is a significant contributor to gaseous toluene and xylene removal, and transported via the stem plays an important role in this process.

## Introduction

The quality of the indoor environment has become a major health consideration in the developed world; a situation exasperated by urban-dweller generally spending 80–90 % of their time in indoors (Krzyanowski 1999; Wang et al. 2007). The quality of indoor air is of particular concern, with over 200 volatile organic compounds (VOCs) having been detected as contaminants (Kostiainen, 1995). Although each compound is likely to be present in very low concentrations, the mixture can produce additive and possibly synergistic effects (Weschler and Shields 1997; World Health Organization 2000). Indoor air is almost 5–10 times more polluted compared to the outdoor environment (Brown et al. 1994; Environment Australia 2003). VOCs such as toluene and xylene are highly toxic and are thought to be significant contributors to reduce indoor air quality (IAQ)-associated health problems and contributing to sick-building syndrome and/or building-related illness (Abbritti and Muzi 1995; Wallace 2001; Orwell et al. 2006; Tsai et al. 2012).

It is well established that “indoor” plants can improve the indoor air quality by reducing many components including volatile organic compounds, such as formaldehyde (Kim et al. 2010; Aydogan and Montoya 2011), benzene (Irga et al. 2013; Torpy et al. 2013), toluene (Oyabu et al. 2005; Kim et al. 2012), and xylene (Wolverton and Wolverton 1993; Kim et al. 2014), thus reducing the risk of sick building syndrome (Kim et al. 2011).

Several reports indicated that microorganisms found in the growing media of indoor potted plants are the primary agents involved in the removal of air-borne VOCs (Orwell et al. 2004; Wood et al. 2006; Chun et al. 2010). This is supported by the fact that the removal of VOC continues to decrease when plant(s) are removed from the media (Wood et al. 2002; Godish and Guindon 1989). A number of soil microorganisms are capable of degrading toxic chemicals (Wenzel 2009), although many of the microbes that are directly associated with VOC removal have not been identified. Wolverton and Wolverton (1993) proposed that the plant leaves absorb formaldehyde and xylene from the air and translocate them via the phloem/xylem to the plant roots where they are degraded by the microorganisms. In the biodegradation pathways of toluene and xylene, 3-methylcatechol by the microorganisms is an intermediate product, then cleavage of the ring of 3-methylcatechol by dioxygenase enzyme and conversion to a non-aromatic compound and CO_2_, subsequently undergo C1 metabolism (Ralph et al. 2006). However, the metabolic pathways for the biodegradation of BTX have been reported earlier (Tsao et al. 1998).

The ratio of the VOC removal via the above ground plants versus directly by the root zone vary for indoor phytoremediation. In our previous study, we showed the ratio of formaldehyde removed by aerial plant parts versus the root zone (in both *Fatsia japonica* and *Ficus benjamina*) was 1:1 during the day, but declined to 1:11 at night when the stomata are closed (Kim et al. 2008). Recently, Kim et al. (2014) reported that the efficiency of volatile toluene and xylene removal by foliage plants was affected by the root zone media volume. Wolverton and Wolverton (1993) showed that top to media VOC removal ratio also varied with plant species and the VOC in question. It was also reported (Wolverton and Wolverton 1993) that during the day, *Dieffenbachia seguine* and *Nephrolepis exaltata* had similar ratios (1:1 aerial plant parts/the root zone) for xylene, whereas the ratio for formaldehyde favored the root zone (37:63 *Dieffenbachia* sp. to 40:60 *Aglaonema* sp.).

However, for the improvement of indoor air quality by means of using indoor potted plants, the present work was designed to investigate the removal efficiency as well as the removal ratios of toluene and xylene (via the stem to root zone versus by direct diffusion from the air into the medium) using foliage plants *Schefflera actinophylla* and *Ficus benghalensis*, and has obtained a considerable decline of toluene and xylene content from the test chamber. Reduction of VOCs levels especially in indoor environment with a low cost environment-friendly potential system may play a vital role to improve human health.

## Materials and methods

### Plant materials

Single stem plants, *S. actinophylla* and *F. benghalensis*, that can easily wrap both shoot and root zone were obtained from a commercial market and used for test. The plants were transplanted into pots containing a uniform growing medium [i.e., Mix #4 (Sun Gro Horticulture, Bellevue, WA), bark-humus (Biocom. Co., Seoul, Korea), and sand at 5:1:1, *v*/*v*/*v*]. Mix #4 contained Canadian sphagnum peat moss (55 to 65 % by volume), perlite, dolomitic lime, gypsum, and a wetting agent. All plants were grown in a greenhouse for 2 weeks after transplanting, and then acclimated within the indoor environment (23 ± 2 °C) for more than 1 month (Kim et al. 2008, 2010). The plants were watered every 3 days with the excess water allowed to drain. All plants were watered 2 days before the gas treatment. Three pots (19-cm i.d. with media volume of 2.2 L) were placed in a chamber with a light intensity of 20 ± 2 μmol·m^−2^·s^−1^ using fluorescent lights over a 24-h period. Six replicates were tested for each treatment. Chambers without plants were used to determine VOC losses not due to the plants (e.g., leakage, adsorption, chemical reactions). The root volume was measured using water displacement in a graduated cylinder; 2 L cylinder was watered up to 1.4 L and was put in the roots, and increased graduation was read (Kim et al. 2014). Plant height and weight were also measured and leaf area determined using a LI-3100 leaf area meter (LI-COR Inc., Lincoln, NE) at the end of the experiment.

### Treatment system

The treatment system consisted of controlled-environment rooms (i.e., temperature, light intensity, and relative humidity) containing the test chamber and a gas generator. The structure of test chamber described by Kim et al. (2011) was 1.0 m^3^ (90 × 90 × 123 cm) and impervious to VOCs. Interior air was circulated (6 L·min^−1^) and tested for toluene and xylene concentration.

### Gas exposure and measurement

Liquid toluene (Sigma-Aldrich Co., Inc., USA) and xylene (Duksan pure chemical Co., LTD., Ansan, Korea) were transformed to gaseous state by a generator machine and were used for pretreatment of the plants, which is known to enhance their phytoremediation potential for each gas (Kim et al. 2008). Gaseous toluene and xylene were introduced in the chamber by a quantitative pump (MP-∑; Sibata Co., Tokyo, Japan) and allowed to equilibrate for 15 min (Kim et al. 2010). To check the leakage of the Teflon bags, an empty chamber (without plant) was used. Three Teflon bags containing ambient fresh air were tightly bound along with air sampling tube, carefully raped with PARAFIM “M,” and one bag was placed outside the chamber and other two bags placed inside the empty chamber. Then toluene and xylene were introduced in the empty chamber, and air sampling from the Teflon bags (both outside and inside chamber) did not detected any toluene and xylene by gas chromatography–mass spectrometry (GC/MS) analysis after 24 h, which confirmed that Teflon bag can exclude VOC. Three potted plants were placed in the test chamber for each treatment. The internal concentration was about 0.5 μL·L^–1^ for the stimulation treatment. The plants remained in the stimulation treatment over a 24-h period and then were moved to fresh air for 2 h. After the stimulation treatment, the subsequent toluene and xylene removal by the plants was determined. The plants were exposed with gas mixing 0.5 μL∙L^−1^ of toluene with 0.3 μL∙L^−1^ of xylene in a chamber. VOC removal was measured within the chamber over a 24-h period with an interval of 3 h. Changes in VOC within the chamber were expressed as concentration (μg·m^−3^) and as removal efficiency on a leaf area (LA) basis (μg·m^−3^·m^−2^ leaf area).

### Air sampling system

**Fig. 1:**
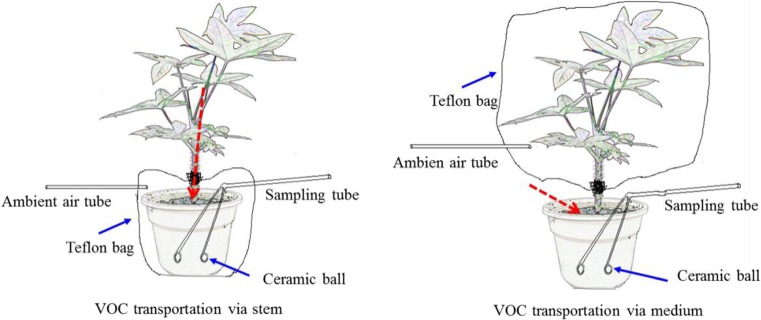
Schematic diagram sampling toluene and xylene which were transported from air in a chamber (1.0 m^3^) to root zone via the stem and by direct diffusion from the air into the medium (*red dashed line* denotes toluene and xylene transporting route via shoot to medium, and direct to the medium, respectively)

Three potted plants were placed in a chamber: one shoot wrapped, one root zone wrapped, and the other one non-wrapping were used for air sampling. For shoot-wrapped, the portion of plant above the medium was sealed with a Teflon bag to determine VOC removal transported by direct diffusion from the air into the medium, and for root zone-wrapped, the belowground portion of plant below the medium was also wrapped to quantify VOC removal via the stem to root as described by Kim et al. (2008; Fig. 1). Ceramic balls were used for sampling of gaseous VOC in the root zone. Ceramic balls were 25 mm i.d. and two ceramic balls per pot plant were buried in 10 cm deep in the medium. Ceramic balls were connected with the sampling tube and also extended the connection to GC/MS through a quartz cold trap. Teflon bags were linked up ambient fresh air through a tube (Kim et al. 2008).

### VOC quantification

Air samples were collected at the appropriate time intervals with the quartz cold trap [120 mm long, 2.9 mm o.d., 1.0 mm i.d. (inlet), 2.0 mm i.d. (outlet); Markes International Ltd., Llantrisant, UK] connected to each chamber and the air was collected for 5 min at 5 mL·min^–1^. An automated thermal desorption system with Air Server autosampler (UNITY; Markes International Ltd.) was connected to the injection port of the gas chromatograph–mass spectrometer (TRACE DSQ; Thermo Electron Co., Waltham, MA) (Kim et al. 2008, 2011). The desorbed sample was cryofocused at 5 °C for 5 min on the first few centimeters of the column, desorbed at 280 °C, and separated using a ZB-624 capillary column (30-m length × 0.25-mm i.d., 1.40-μm film thickness of 6 % cyanopropylphenyl, 94 % dimethylpolysiloxane; Phenomenex, Torrance, CA). The injection port temperature was 180 °C with a split ratio of 29:1. Helium was used as the carrier gas at a flow rate of 1.0 mL·min^–1^. The column temperature was held at 45 °C for 1 min and increased at a rate of 15 °C·min^–1^ to 100 °C and held for 1 min and then increased at a rate of 5 °C·min^–1^ to 135 °C. However, the transfer line temperature was 180 °C; the ion source temperature was 280 °C. The mass range was *m*/*z* 45–120 and the mode of detection was EI mode (70 eV).

### Data analysis

Gas concentrations were expressed as microgram per cubic meter with the data normalized to 24 ± 1 °C and 100 kPa (Hines et al. 1993). Data were expressed as the average of six replicates. The concentration of toluene or xylene [Eq. 1] and the removal efficiency per unit leaf area and time [Eq. 2] were calculated (Kim et al. 2008, 2011) as:

1 $$ \mathrm{V}\mathrm{O}\mathrm{C}\ \mathrm{concentration}\ \left(\upmu \mathrm{g}\cdotp {\mathrm{m}}^{-3}\right) = \left[\left(\mathrm{Pi} - \left(\mathrm{Ci} - \mathrm{C}\right)\right) - \mathrm{P}\right] \times \left(\mathrm{F} \times \mathrm{C}\mathrm{V}\right) $$

2 $$ \mathrm{V}\mathrm{O}\mathrm{C}\ \mathrm{removal}\ \left(\upmu \mathrm{g}\cdotp {\mathrm{m}}^{-3}\cdot {\mathrm{m}}^{-2}\mathrm{leaf}\ \mathrm{area}\right) = \left[\left(\mathrm{Pi}-\left(\mathrm{Ci}-\mathrm{C}\right)\right)-\mathrm{P}\right] \times \left(\mathrm{F} \times \mathrm{C}\mathrm{V}\right)/\mathrm{L} $$

where P is the gas concentration measured in a chamber with plants (μL·L^–1^); Pi the initial gas concentration measured in a chamber with plants (μL·L^–1^); C the gas concentration measured in a chamber without plants (μL·L^–1^); Ci the initial gas concentration measured in a chamber without plants (μL·L^–1^); F the toluene or xylene conversion factor for volume (μL·L^–1^) to mass (mg·m^–3^); CV the volume of the chamber (m^3^); and L the total leaf area per chamber (m^2^). The loss of toluene or xylene (Ci − C) not resulting from the plant and media was determined using empty chambers.

## Results

**Fig. 2:**
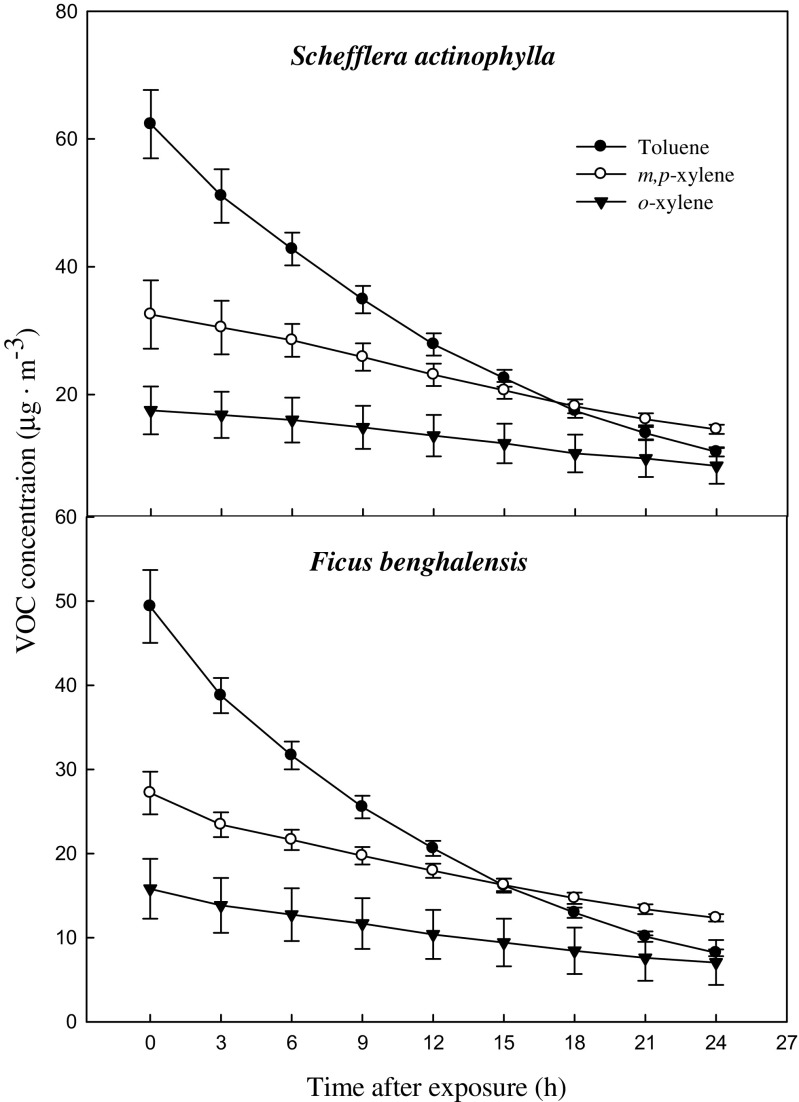
Decline of gaseous toluene and xylene caused by potted *S. actinophylla* and *F. benghalensis*. Three potted plants were exposed for 24 h in a sealed chamber (1.0 m^3^) at a light intensity of 20 ± 2 μmol·m^−2^·s^−1^. *Vertical bars* denote the SE (*n* = 6)

**Fig. 3:**
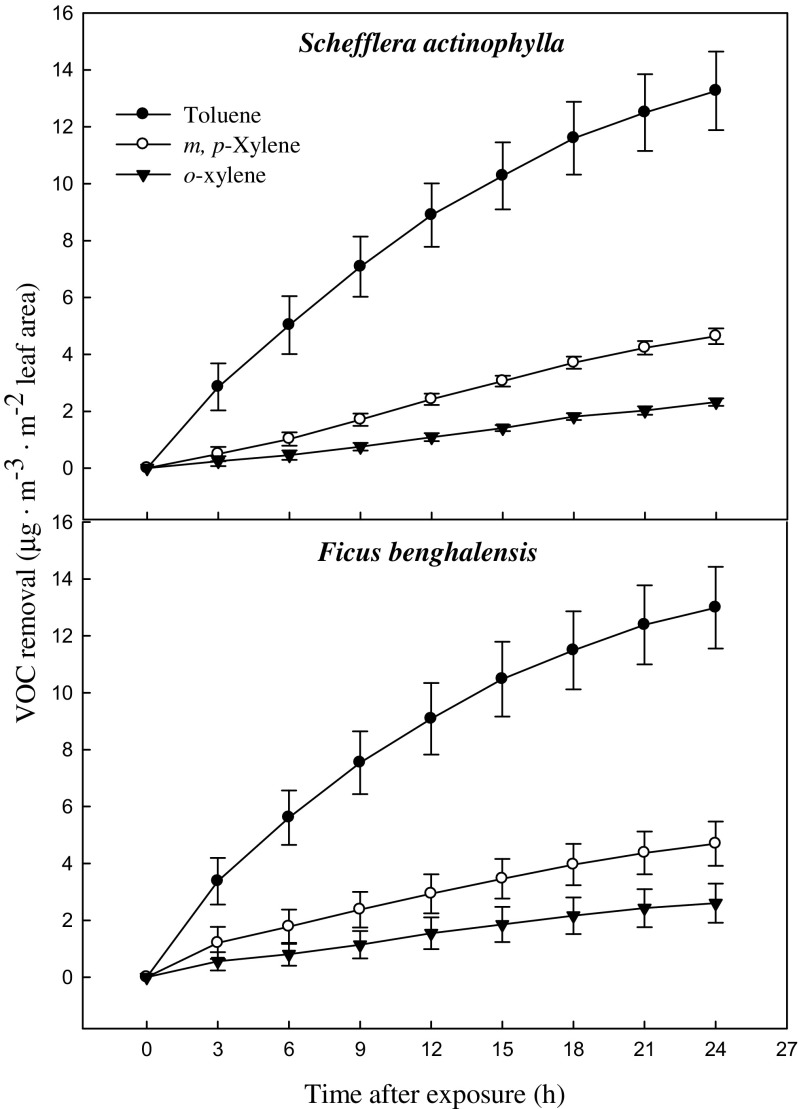
Removal efficiency of gaseous toluene and xylene by potted *S. actinophylla* and *F. benghalensis*. Three potted plants were exposed for 24 h in a sealed chamber (1.0 m^3^) at a light intensity of 20 ± 2 μmol·m^−2^·s^−1^. *Vertical bars* denote the SE (*n* = 6)

The characteristics of the test plants (*S. actinophylla* and *F. benghalensis*) are shown in Table 1. Due to different root systems, there was a big difference in root volume and root weight among two species. The leaf area were 0.20, and 0.16 m^3^/pot for *S. actinophylla* and *F. benghalensis*, respectively. Gaseous toluene and xylene were removed by indoor potted plants, *S. actinophylla* and *F. benghalensis* in the sealed chamber (Fig. 2). Toluene and xylene concentrations in the chamber were continuously decreased with time by plants. Toluene declined sharply, while xylene decreased slowly in both species. Liquid xylene (Duksan pure chemical Co., LTD., Ansan, Korea) was volatilized and separated into three isomeric *m-*, *p-*, and *o*-xylene. However, *m-* and *p*-xylene were detected at the same retention time of GC/MS, and each of them could not be quantifiable. Initial concentration of toluene and total xylene was 62.3 and 49.4 μg·m^−3^ in *S. actinophylla*, and was 50.1 and 43.0 μg·m^−3^ in *F. benghalensis*, respectively. Removal efficiency of toluene and total xylene (*m*, *p*, *o*) was 13.3 and 7.0 μg·m^−3^·m^−2^ leaf area over a 24-h period, and was 0.55 and 0.29 μg·m^−3^·h^−1^·m^−2^ leaf area when converted into per hour in *S. actinophylla* (Fig. 3). In *F. benghalensis*, removal efficiency of toluene and total xylene was 13.0 and 7.3 μg·m^−3^·m^−2^ leaf area over a 24-h period, and efficiency per hour was 0.54 and 0.31 μg·m^−3^·h^−1^·m^−2^ leaf area, respectively. Removal efficiency of toluene was high compared with total xylene, and *m*,*p*-xylene was removed more than *o*-xylene in both species.

**Table 1:** Characteristics of plants used for test

Species	Plant height (cm)	Root		Leaf area (m^3^/pot)
		Volume (mL/pot)	Fresh weight (g/pot)	
*Schefflera actinophylla*	48.7 ± 1.57	17.8 ± 4.8	4.3 ± 1.0	0.20 ± 0.01
*Ficus benghalensis*	58.2 ± 2.35	66.7 ± 8.9	63.0 ± 8.4	0.16 ± 0.01

**Fig. 4:**
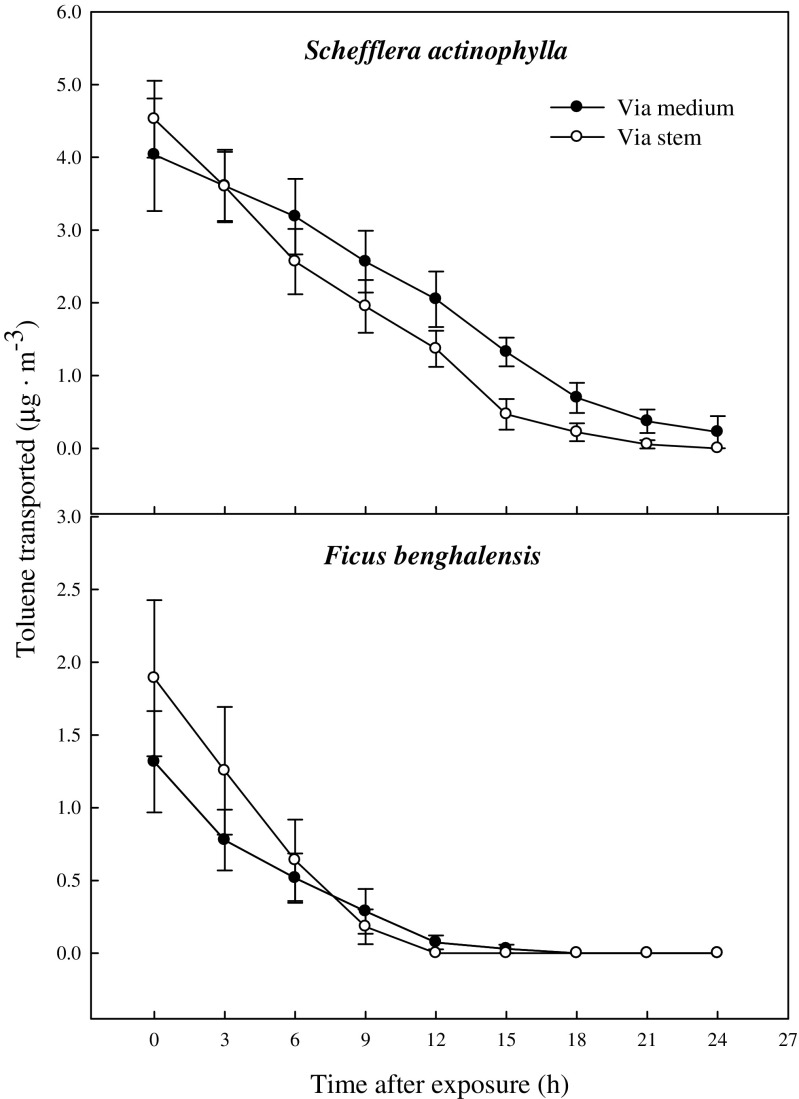
Gaseous toluene transported from air to root zone: via the stem to root and by direct diffusion from the air into the medium of potted *S. actinophylla* and *F. benghalensis*. Gaseous toluene was sampled inside medium of pot plants wrapping shoot or root zone. Three potted plants were exposed for 24 h in a sealed chamber (1.0 m^3^) at a light intensity of 20 ± 2 μmol·m^−2^·s^−1^. *Vertical bars* denote the SE (*n* = 6)

Although shoot or root zone was wrapped not to contact with gas exposed in the chamber, lots of gaseous toluene and xylene were detected in the middle of the plants medium. This is indicating that VOC in air is absorbed through leaf and transported via the stem, and finally reached to root zone. Toluene and xylene transported to root zone via both stem and medium were decreased with decline of their concentration of circumferential air in the chamber (Figs. 1 and 4). At first, toluene concentration transported to root zone via the stem was higher than that of direct diffusion from the air into the medium, but reversed at 3 h after exposure in *S. actinophylla* and at 9 h in *F. benghalensis* (Fig. 4). After 15 h, toluene was very little detected in the middle of the plants medium, and toluene via the stem was not found at 24 h after exposure in *S. actinophylla*, and at 12 h in *F. benghalensis*.

**Fig. 5:**
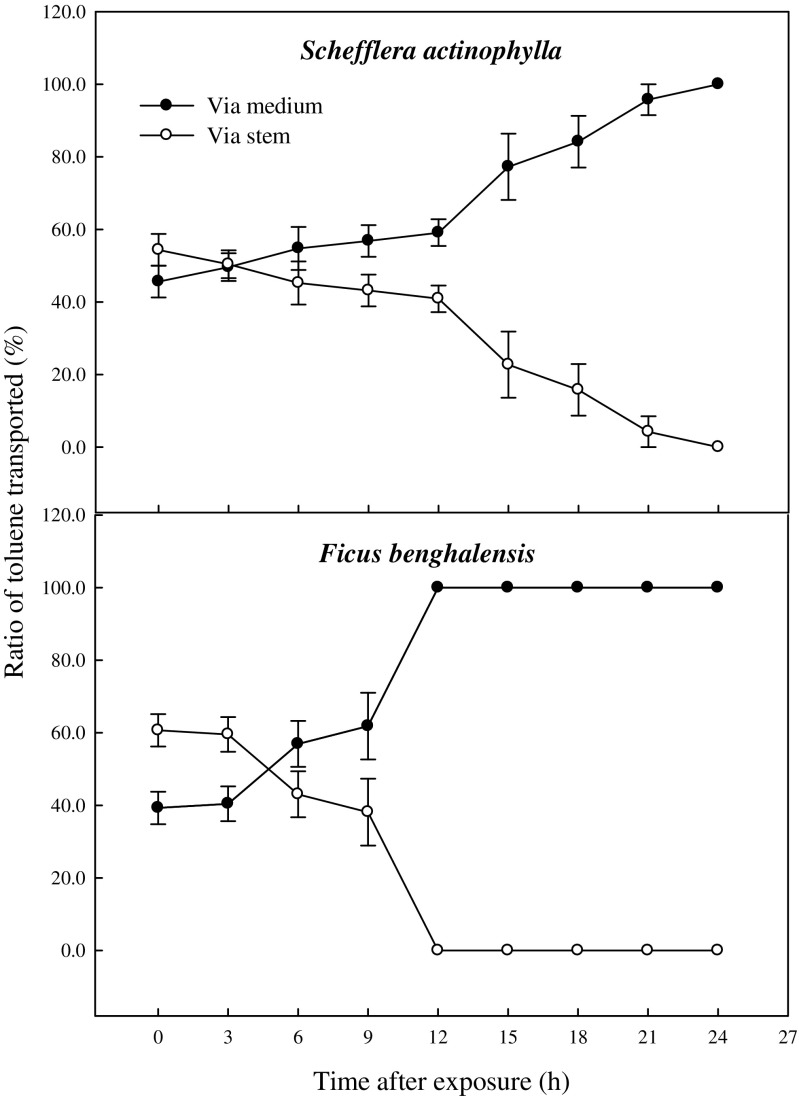
Comparison of gaseous toluene ratio transported from air to root zone: via the stem to root (S) and by direct diffusion from the air into the medium (M) (i.e., S or M/(S + M) × 100 %) of potted *S. actinophylla* and *F. benghalensis*. Gaseous toluene was sampled inside medium of pot plants wrapping shoot or root zone. Three potted plants were exposed for 24 h in a sealed chamber (1.0 m^3^) at a light intensity of 20 ± 2 μmol·m^−2^·s^−1^. *Vertical bars* denote the SE (*n* = 6)

**Fig. 6:**
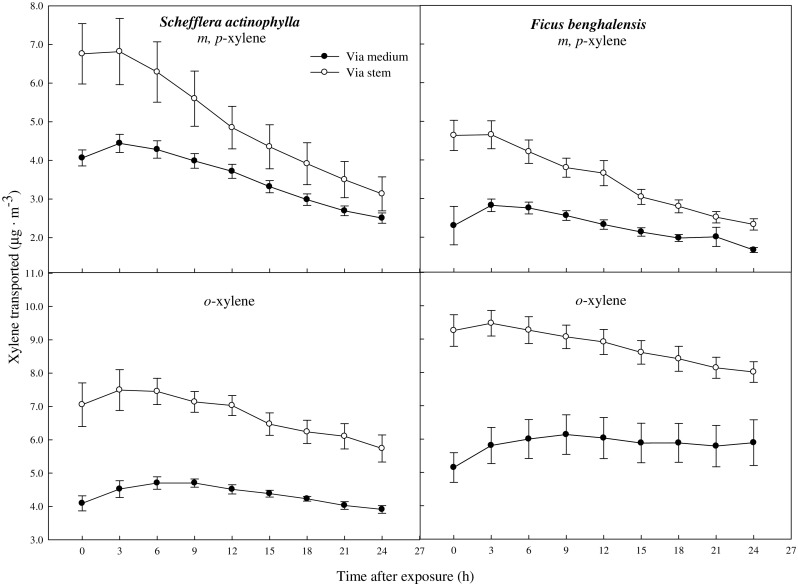
Gaseous xylene transported from air to root zone: via the stem to root and by direct diffusion from the air into the medium of potted *S. actinophylla* and *F. benghalensis*. Gaseous xylene was sampled inside medium of pot plants wrapping shoot or root zone. Three potted plants were exposed for 24 h in a sealed chamber (1.0 m^3^) at a light intensity of 20 ± 2 μmol·m^−2^·s^−1^. *Vertical bars* denote the SE (*n* = 6)

**Fig. 7:**
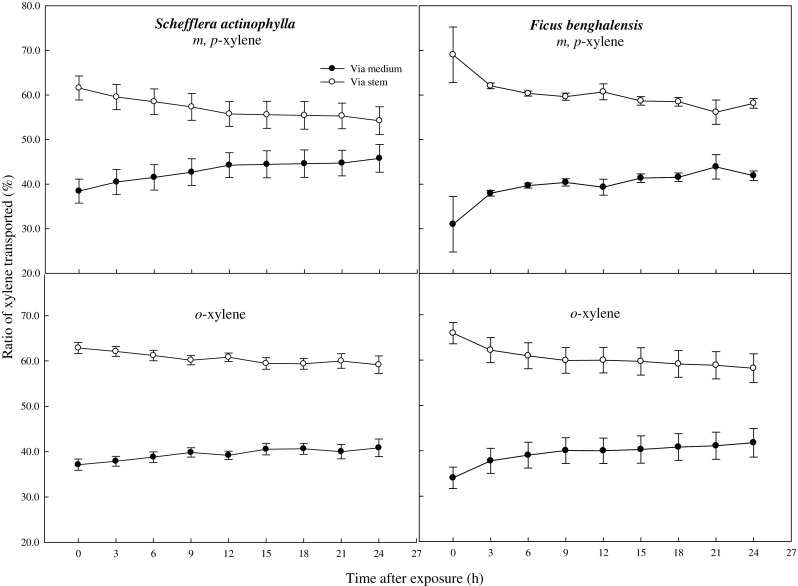
Comparison of gaseous xylene transported from air to root zone: via the stem to root (S) and by direct diffusion from the air into the medium (M) (i.e., S or M/(S + M) × 100 %) of potted *S. actinophylla* and *F. benghalensis*. Gaseous xylene was sampled inside medium of pot plants wrapping shoot or root zone. Three potted plants were exposed for 24 h in a sealed chamber (1.0 m^3^) at a light intensity of 20 ± 2 μmol·m^−2^·s^−1^. *Vertical bars* denote the SE (*n* = 6)

**Fig. 8:**
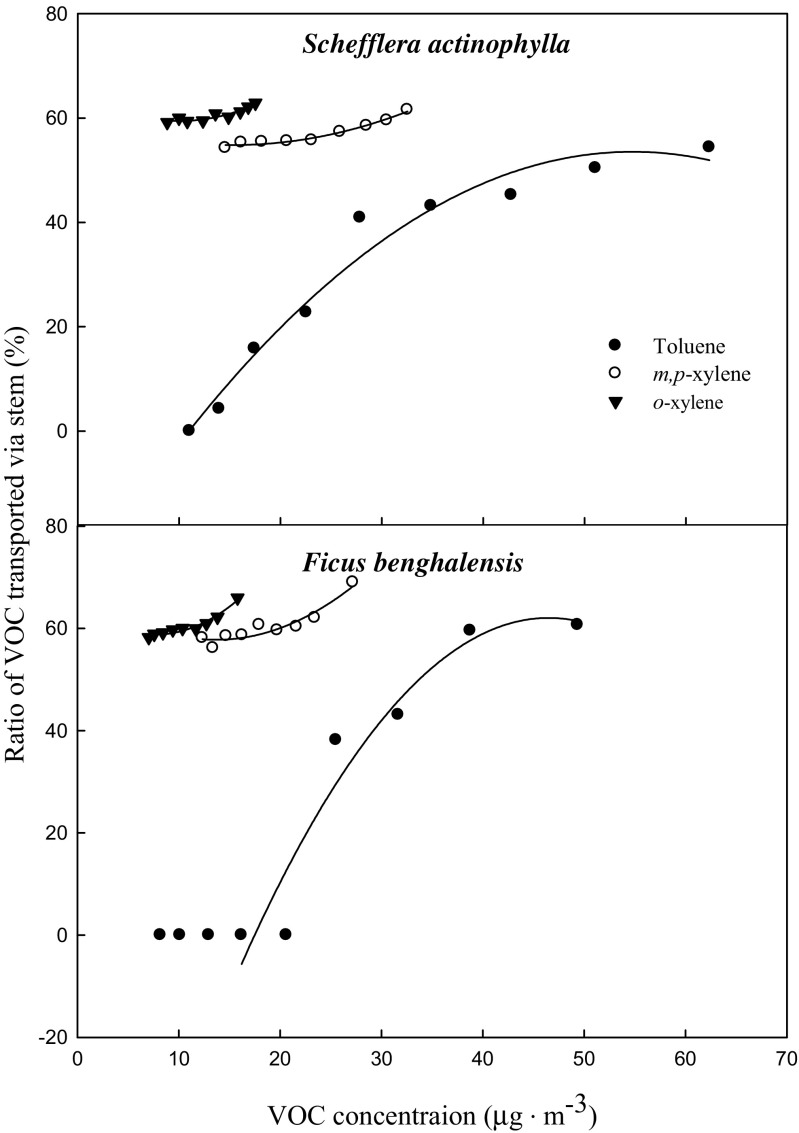
Gaseous toluene and xylene ratio transported from air to root zone via the stem according to VOC concentration. Gaseous toluene and xylene were sampled inside medium of pot plants wrapping root zone. Three potted plants were exposed for 24 h in a sealed chamber (1.0 m^3^) at a light intensity of 20 ± 2 μmol·m^−2^·s^−1^. *Vertical bars* denote the SE (*n* = 6)

The ratio of toluene transported via the stem was decreased with time after exposure (Fig. 5). The ratios of initial toluene transported via the stem and by direct diffusion from the air into the medium were 54.4 and 45.6 % in *S. actinophylla*, and 60.7 and 39.3 % in *F. benghalensis*, respectively. The ratio of toluene transported via the stem was similar with that by direct diffusion from the air into the medium after 3 h, and it was gradually decreased up to 0 % in *S. actinophylla*. After 12 h, the toluene was little detected in the middle of the medium, even though ratio transported had big difference between via the stem and by direct diffusion from the air into the medium in both species (Figs. 4 and 5). Xylene transported via the stem was higher than that of direct diffusion from the air into the medium (Fig. 6). Initial concentration of xylene transported was lower, and increased a little up to 3 or 6 h, and then diminished. The ratios of xylene transported via the stem were also decreased, but that by direct diffusion from the air into the medium was increased (Fig. 7). Even though the ratio of xylene via the stem was decreased, it was higher than that by direct diffusion from the air into the medium over a 24-h period. The result for gaseous toluene and xylene ratio transported from air to root zone via the stem showed that when toluene present at high concentration in the test chamber then transportation percentage was increased, whereas *m*,*p*-xylene and *o*-xylene transportation percentage via the stem to root zone was increased even at low concentration in *S. actinophylla* (Fig. 8). Similar pattern was also observed for toluene and xylene transportation via the stem to root zone in *F. benghalensis*.

## Discussion

Poor indoor air quality is a worldwide problem with tremendous human health and economic consequences. Hence, there is a need for designing specific and suitable air-purifying system to clean the indoor air of affected buildings. Reports are available on the removal of VOCs from the indoor air by potted plants (Orwell et al. 2004; Burchett et al. 2008; Yang et al. 2009; Kim et al. 2010, 2012).

Mosaddegh et al. (2014) reported that *Opunita microdasys* was able to remove 2 ppm concentration of toluene and xylene singly from air in the test chambers completely after 55 and 47 h respectively, whereas *Dracaena dermensis* could remove 2 ppm of toluene and xylene singly from air in the test chambers completely after 120 and 98 h, respectively. The removal rate of toluene from air in the test chambers was 0.54 and 0.24 mg·m^−3^·day^−1^ for *O. microdasys* and *D. dermensis*, respectively, based on leaf area. On the other hand, the removal rate of xylene from air in the test chambers was 1.64 and 0.76 mg·m^−3^·day^−1^ for *O. microdasys* and *D. dermensis*, respectively, based on leaf area.

However, in the present study, we used gaseous toluene and xylene mixture, and initial concentration of toluene and total xylene was 62.3 and 49.4 μg·m^−3^, respectively, in *S. actinophylla*, and was 50.1 and 43.0 μg·m^−3^ in *F. benghalensis*. Gaseous toluene and xylene removal per hour were 0.55 and 0.29 μg·m^−3^·h^−1^·m^−2^ leaf area in *S. actinophylla*. In *F. benghalensis*, toluene and total xylene removal per hour were 0.54 and 0.31 μg·m^−3^·h^−1^·m^−2^ leaf area. Removal efficiency of toluene was high compared with total xylene, and *m*,*p*-xylene was removed more than *o*-xylene in both species. Similar pattern of toluene removal efficiency per hour from air in the test chamber based on leaf area was reported by Mosaddegh et al. (2014).

The ratio of VOC removal by via shoot to root zone versus via direct medium is a critical process of phytoremediation. Wolverton and Wolverton (1993) reported that the ratios of VOC removal varied with plant species. In our previous study, we observed the average ratio of formaldehyde removed by aerial plant parts versus the root zone (in both *F. japonica* and *F. benjamina*) was 1:1 (52 and 48 %) during the day but declined to 1:11 at night when the stomata are closed (Kim et al. 2008). The effectiveness of the root zone in formaldehyde removal was due preliminarily to microorganisms and roots (∼90 %); only about 10 % was due to adsorption by the growing medium. This result indicated that the root zone is a major contributor to removal of formaldehyde (Kim et al. 2008).

It is assumed that at the initial stage, the concentration of toluene and xylene in the test chamber may remain unstable or not in uniform condition. At the same time, the acceptability of plants to absorb VOCs and transported to root zone via the stem or by direct diffusion from the air into the medium may also vary initially. The concentration of toluene and xylene normally become low in the test chamber at the late hours of exposure to plants (Fig. 1). So the uniformity of the transportation rate of toluene and xylene from air to root zone via the stem, and by direct diffusion from the air into the medium, 3- to 9-h time duration was considered to calculate the rate of transportation of toluene and xylene in this study. As a result, the ratios of toluene transported via the stem and by direct diffusion from the air into the medium were 46.3 and 53.7 % in *S. actinophylla*, and 46.9 and 53.1 % in *F. benghalensis*, and then about 47 and 53 % on averages of both species. The ratios of *m*,*p*-xylene transported via the stem and by direct diffusion from the air into the medium were 58.5 and 41.5 % in *S. actinophylla*, and 60.7 and 39.3 % in *F. benghalensis*, and then about 60 and 40 % in both species. The ratio of *o*-xylene transported via the stem and by direct diffusion from the air into the medium was 61 and 39 % in both species, so the ratios of total xylene were about 60 and 40 %.

Plants are known to absorb air pollutants through their stomata, cuticle, or epidermis, and then translocate them to their root zone via the stem where an abundance of microbes thrives, and pollutants are broken down by the microorganisms (Wolverton and Wolverton 1993). A number of plants have been reported to absorb through leaf and metabolized air-borne VOCs such as benzene and toluene (Cornejo et al. 1999; Wood et al. 2002), toluene and xylene (Oyabu et al. 2005; Kim et al. 2012; Kim et al. 2014), and benzene, toluene, and xylene (Chun et al. 2010).

Transportation of VOCs via the stem to root zone depends on the concentration and the type of VOCs in the indoor spaces or test chamber, although VOC is absorbed and metabolized by both leaves and the rhizosphere microorganisms (Orwell et al. 2004). In the present study, the transportation rate of toluene and xylene via the stem to root zone was increased with increasing the concentration of toluene in the chamber in both *S. actinophylla* and *F. benghalensis* even if the transportation rate of xylene via the stem to root zone was high at low concentration of xylene present in the test chamber (Fig. 8). It is assumed that when the concentration of toluene is low in the chamber, plant leaves are metabolized most of the amount and the less amount transported to the root zone via the stem. On the other hand, when toluene concentration is high in the chamber, then leaves are not enough to degrade and maximum percent of toluene transported to root zone via the stem. After entering the leaf, a compound can undergo degradation, storage, or excretion, either at the site of uptake or after translocation to other parts of the plant (Cruz et al. 2014). Tani and Hewitt (2009) observed that for eight aldehydes and five ketones, the amount of pollutant taken up was 30–100 times higher than what theoretically could be absorbed by the phase inside the leaves. This finding indicated the metabolism or translocation (Tani and Hewitt 2009). The VOC uptake and translocation pathway by the aboveground plant is mostly dependent on the properties of VOCs. A hydrophilic VOC such as formaldehyde will not diffuse easily through the cuticle that consists of lipids, whereas a lipophilic VOC such as benzene is more likely to penetrate through the cuticle (Cruz et al. 2014). *Glycene max* cells were exposed to ^14^C labeled formaldehyde, and the allocation of the ^14^C indicated that formaldehyde was firstly detoxified by oxidation and subsequently underwent C1 metabolism (Giese et al. 1994).

During the biodegradation process, the concentration of VOCs in the micro-environment where microorganisms are found has a profound impact on microbial activity, and ultimately on the pollutant removal rate. Daisey et al. (1994) reported that gaseous toluene must be transferred into aqueous phase before being biodegraded. However, biodegradation of BTX hydrocarbons has been shown in two principle aerobic pathways: a dioxigenase attack on the aromatic ring, referred to as the *tod* pathway, and a monooxigenase attack on methyl substituent, referred to as the *tol* pathways (Mikesell et al. 1993). Benzene can be metabolized by the *tod* pathway only, but toluene and xylene may be subjected to oxidation by either *tod* or *tol* pathway. It has been shown that in the case of *p*-xylene, the *tod* pathway can lead to a dead-end product, 3,6-dimethylcatechol (Gibson et al. 1974), and an analogous transformation was observed in the metabolism of *p*-xylene and *o*-xylene by *Pseudomonas putida* (Oh et al. 1994).

In addition, the ability to metabolize VOCs varies widely among plant species and volatiles compounds (Cape 2003). Therefore, a better understanding of the basic physical and chemical factors modulating the phytoremediation processes in the most efficient species is needed. Maximizing the VOC removal efficiency of plants will help to expand the range of situation in which plants can be used effectively.

To the best of our knowledge, there is no report available on the removal of gaseous toluene and xylene ratio transported via the stem, and by direct diffusion from the air into the medium by indoor plants. Further studies are necessary for understanding this phenomenon.

## Conclusions

The results of the present study confirmed that both *S. actinophylla* and *F. benghalensis* plants reduced toluene and xylene from the test chamber. In both species, average toluene transported ratio via the stem and by direct diffusion from the air into the medium was 47 and 53 %, and the ratios of *m*,*p*-xylene transported was 60 and 40 %. The ratio of *o*-xylene transported via the stem and by direct diffusion from the air into the medium was 61 and 39 % in both species. In conclusion, this result suggested that for gaseous toluene and xylene removal, root zone is the potential contributor, and being transported via the stem plays an important role. Both *S. actinophylla* and *F. benghalensis* can be used as indoor plants to remove the VOCs especially toluene and xylene.
